# Second look Holter ECG in neurorehabilitation

**DOI:** 10.1186/s42466-019-0046-9

**Published:** 2019-12-20

**Authors:** Stefan Knecht, Sebastian Petsch, Paulus Kirchhof, Bettina Studer

**Affiliations:** 1 Mauritius Hospital, Strümper Straße 111, 40670 Meerbusch, Germany; 2 Institute of Clinical Neuroscience and Medical Psychology, Medical Faculty, Heinrich-Heine-University, Düsseldorf, Germany; 3 University of Birmingham, Centre of Cardiovascular Sciences and SWBH NHS trust, Birmingham, UK; 4 Department of Cardiovascular Medicine, University Hospital Münster, Münster, Germany and AFNET, Münster, Germany

**Keywords:** Atrial fibrillation, Stroke, Neurorehabilitation, Holter ECG

## Abstract

**Background:**

Many stroke survivors suffer recurrent stroke because paroxysmal atrial fibrillation (AF) was missed and no preventive anticoagulation initiated. This prospective cohort study determined the added diagnostic yield of second-look 24-h electrocardiographic recording (ECG) in a population at high risk for AF: patients who suffered a stroke of such severity that they require inpatient neurorehabilitation.

**Methods:**

We enrolled 508 patients with ischemic stroke admitted to post-acute inpatient neurorehabilitation and determined whether AF was detected during acute care at the referring hospital. Second-look baseline and 24-h Holter ECG were then conducted during neurorehabilitation. Primary outcome was number of newly detected AF with duration of > 30 s; secondary outcomes were number of newly detected absolute arrhythmia of 10–30 s and < 10 s duration. For comparison, we further enrolled 100 patients with hemorrhagic stroke without history of AF (age = 72 + 11 years, 51% female).

**Results:**

In 206 of the 508 ischemic stroke patients, AF had been detected during acute phase work-up (age = 78 + 10 years, 55% female). For the remaining 302 ischemic stroke patients, no AF was detected during acute phase work-up (age = 74 + 9 years; 47% female). Second-look 24-h ECG showed previously missed AF of > 30 s in 20 of these patients, i.e. 6.6% of the sample, and shorter absolute arrhythmia in 50 patients (i.e. 16.5%).

**Conclusions:**

Second-look 24-Hour ECG performed during post-acute inpatient neurorehabilitation has a high diagnostic yield and should become a standard component of recurrent stroke prevention.

## Introduction

Atrial fibrillation (AF) is the leading preventable cause of recurrent stroke but easily escapes detection when paroxysmal. Moreover, short length of stay in acute stroke care adds to the diagnostic challenge. As of yet, we have no basis for anticoagulant treatment of patients with embolic stroke of undetermined source without documentation of AF [1–3]. Therefore, detection of AF remains a cornerstone for overall prevention of recurrent stroke. But, it is still unclear who should be monitored, when, and for how long [4]. Current stroke guidelines recommend 24-h electrocardiographic (ECG) monitoring [5, 6]; yet previous research indicates that a considerable portion of AF is missed with this standard work-up [7]. Prolonged continuous ECG monitoring with long-term non-invasive or implantable monitor systems might be a solution [7–10]. However, availability of implantable devices is still limited [11]. The current study investigated the diagnostic yield of a 2nd-look 24-h ECG conducted during post-acute inpatient neurorehabilitation. Stroke survivors who require inpatient neurorehabilitation due to severe functional impairment have a particularly high risk for AF because stroke severity is closely associated with probability of AF [12, 13]. Therefore, in terms of total diagnostic yield and cost, second-look 24-h Holter ECG in high-risk patients could compare favorably with more extensive monitoring in lower-risk populations [14].

Our primary objective was to determine the *added* diagnostic yield of this procedure. Therefore, we prospectively recruited and tested patients with a diagnosis of ischemic stroke for whom no AF was detected during acute care.

A secondary objective was to estimate the total rate of AF in ischemic stroke patients requiring inpatient neurorehabilitation. Therefore, we also recruited and tested patients with a diagnosis of ischemic stroke for whom AF was detected during acute care work-up.

Finally, to rule out that AF resulted from brain lesion rather than being the cause of ischemic strokes, we recruited and tested a comparison group of patients with hemorrhagic stroke (and no history of AF or anticoagulation treatment).

## Methods

The study was performed in a single inpatient neurorehabilitation center (Mauritius Hospital Meerbusch, Germany) with 200 beds serving a catchment area of approximately 2.8 million people. Admission criteria to inpatient neurorehabilitation are substantial functional deficits corresponding to a modified Rankin score of three or worse attributable to acute neurological disease with a potential for improvement.

We prospectively included consecutive patients with ischemic or hemorrhagic strokes over an 18-months period. All survivors of acute ischemic or hemorrhagic stroke admitted to our center after hospital-based acute care were enrolled and recruitment was terminated once the pre-specified recruitment target of 600 patients was met. The number was chosen to compare with other studies in the field [9]. No further distinction was made between stroke etiologies such as lacunar or embolic because the extent of brain damage qualifying for inpatient neurorehabilitation was always compatible with a possible cardiac embolism. Exclusion criteria were primary intracranial hemorrhages with known AF or association with oral anticoagulation. Patients were assessed for demographics, acute phase (i.e. pre-rehabilitation) work-up, and CHA2DS2VASc scores. Holter ECG was conducted using a commercially available 3-lead Holter monitor device (Custo-med GmbH, Germany). ECG recordings were analyzed by independent expert investigators, and blinded to clinical data using dedicated analysis software (custo-med GmbH for Windows, Version 4.3.1 Build 18,597).

AF was defined as at least one period of greater than 30 s duration of an absolute arrhythmia (AA) without detectable P-waves and without a pattern more consistent with an alternative diagnosis [15]. Additionally, shorter arrhythmias were analyzed and classified as short atrial arrhythmias, i.e. of 10 to 30 s duration, or as atrial runs, i.e. of less than 10 s duration, because atrial arrhythmias of less than 30 s duration have been associated with increased stroke risk and are often used in clinical decision-making [9, 16].

Analysis of variance, chi-square tests, t-tests for independent samples (two-sided, α = .05) and logistic regression were used for statistical analysis, performed in SPSS (Version 22).

### Ethics committee approval

This research was approved by the Independent Ethics Committee of the Heinrich Heine University Düsseldorf, Germany (protocol no 4670).

### Role of the funding source

**Fig. 1:**
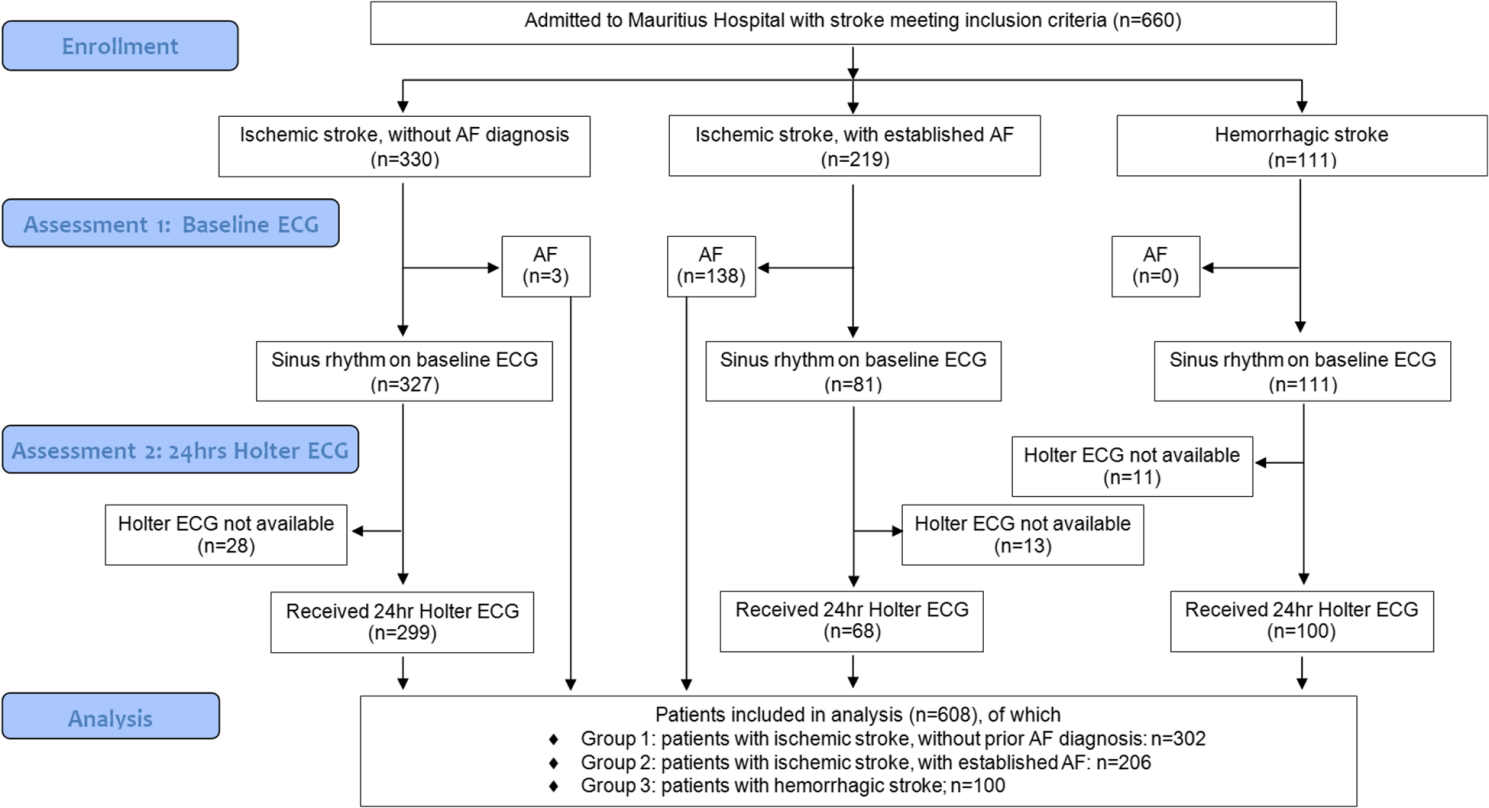
Selection of patients evaluated in the study. Note that ECG assessment was sequential, consisting of a baseline ECG and if negative a Holter ECG

This study was funded by the Mauritius Hospital Meerbusch, a non profit institution. The funder was not involved in the design or conduct of the study or the preparation of this manuscript (Fig. 1).

## Results

### Group classification

A total of 608 patients were entered in the analysis, of which 508 had ischemic stroke and 100 had hemorrhages (=comparison group). During acute care, a diagnosis of AF had been established in 206 of patients with ischemic stroke (i.e. in 40.5% of cases), whereas no AF had been detected during acute care in the remaining 302 of patients with ischemic stroke. The mean latency between stroke and admission to neurorehabilitation was 24 days in both groups with ischemic stroke and 26 days in patients with hemorrhage.

Demographic characteristics, concomitant conditions and cardiovascular risk as estimated by the CHA_2_DS_2_VASc score are displayed in Table 1. Ischemic stroke patients with AF established during acute care were significantly older than ischemic stroke patients without a diagnosis of AF (delta = 3.5 years, LDS, *p* = 0.02) and patients with cerebral hemorrhage (delta = 6.2 years, *p* < .001). Furthermore, ischemic stroke patients with AF established during acute care had a higher total CHA_2_DS_2_VASc score than patients without a diagnosis of AF (delta = 0.15, p = 0.02) or with cerebral hemorrhage (delta = 2.74, *p* < .001). Meanwhile, ischemic stroke patients without AF established during acute care were older (delta = 2.6 years, *p* = .03) and had a higher CHA_2_DS_2_VASc score (delta = 2.5, p < .001) than patients with cerebral hemorrhage.

**Table 1:** Patient characteristics including mean CHA_2_DS_2_VASc -score

Group	Ischemic stroke admitted to neuro-rehabilitation without diagnosis of AF	Ischemic stroke admitted to neuro-rehabilitation with diagnosis of AF	Hemorrhagic stroke	Group comparison
Number of patients	302	206	100	
Mean age + SD	74 + 9	78 + 10	72 + 11	*F* = 15.36 *p* = .001 ^a^
Prevalence of stroke risk factors on CHA_2_DS_2_VASc				
Congestive heart failure	15%	22%	9%	X^2^ = 8.61 *p* = .01 ^b^
Hypertension	95%	98%	86%	X^2^ = 17.52 *P* = .001 ^b^
Age > 75 years	48%	72%	48%	X^2^ = 31.41 *P* = .001 ^b^
Age 65–74 years	37%	20%	31%	X^2^ = 15.20 *P* = .001 ^b^
Diabetes mellitus	21%	22%	17%	X^2^ = 1.02 *P* = .60
Stroke/ TIA/ Thrombo-embolic events	100%	100%	3%	X^2^ = 586.29 *P* = .001 ^b^
Vascular disease	42%	17%	8%	X^2^ = 60.78 *P* = .001 ^b^
Female sex	47%	55%	51%	X^2^ = 3.40 *P* = .18
Mean Total CHA_2_DS_2_VASc –score				
Mean score + SD	5.52 ± 1.28	5.78 ± 1.18	3.03 ± 1.30	*F* = 182.57, *P* = .001 ^c^

^a^ Pairwise post-hoc comparisons revealed hemorrhagic stroke patients were younger than both ischemic stroke patient groups (LSD, ps < .02), and ischemic stroke patients admitted with established AF were significantly older than ischemic stroke patients without AF documented during acute phase work-up (LSD, *p* = .001)

^b^ For *Congestive heart failure*, *Hypertension and Stroke/TIA/Thrombo-embolic events* the group effect was driven by a lower prevalence in the hemorrhagic stroke group compared to the two ischemic stroke groups. For *Vascular disease sex* the group effect was mainly driven by the ischemic group without established AF. For the two *Age-related risk factors* and *Female sex* the group effect was mainly driven by the ischemic group with established AF

^c^ Post-hoc comparisons showed that hemorrhagic stroke patients had a lower score than the two ischemic groups (LSD, ps < .03), and that ischemic stroke patients with no documentation of AF had a lower score than those with established AF (LSD, ps = .02)

### Added diagnostic yield of post-acute second-look 24-h ECG

Second-look baseline ECG during neurorehabilitation found AF in 3 of the 302 patients with ischemic stroke for whom no AF was found during acute-care work-up. Second look 24-h Holter ECG revealed AF in an additional 17 patients of this group. In total, previously undetected AF was thus found in 6.6% of patients with ischemic stroke admitted without a diagnosis of AF. A further 11 patients (i.e. 3.6%) showed absolute arrhythmia of 10–30 s on the 24-h and 39 patients (i.e. 12.9%) showed absolute arrhythmia of < 10 s on the 24-h Holter ECG.

Patients for whom AF was newly detected during this second-look ECG work-up were moderately, but not significantly, older (delta = 3.2 years, *p* = .07) than patients for whom AF was not detected in either the acute phase nor on second-look ECG. Total CHA_2_DS_2_VASc score (delta = .29, *p* = .32) and prevalences of the individual risk factors comprised in the CHA_2_DS_2_VASc score did not differ between the two groups (all X^2^ < 1.45, *p* > .25).

### Total rate of AF in ischemic stroke patients requiring inpatient neurorehabilitation

**Fig. 2:**
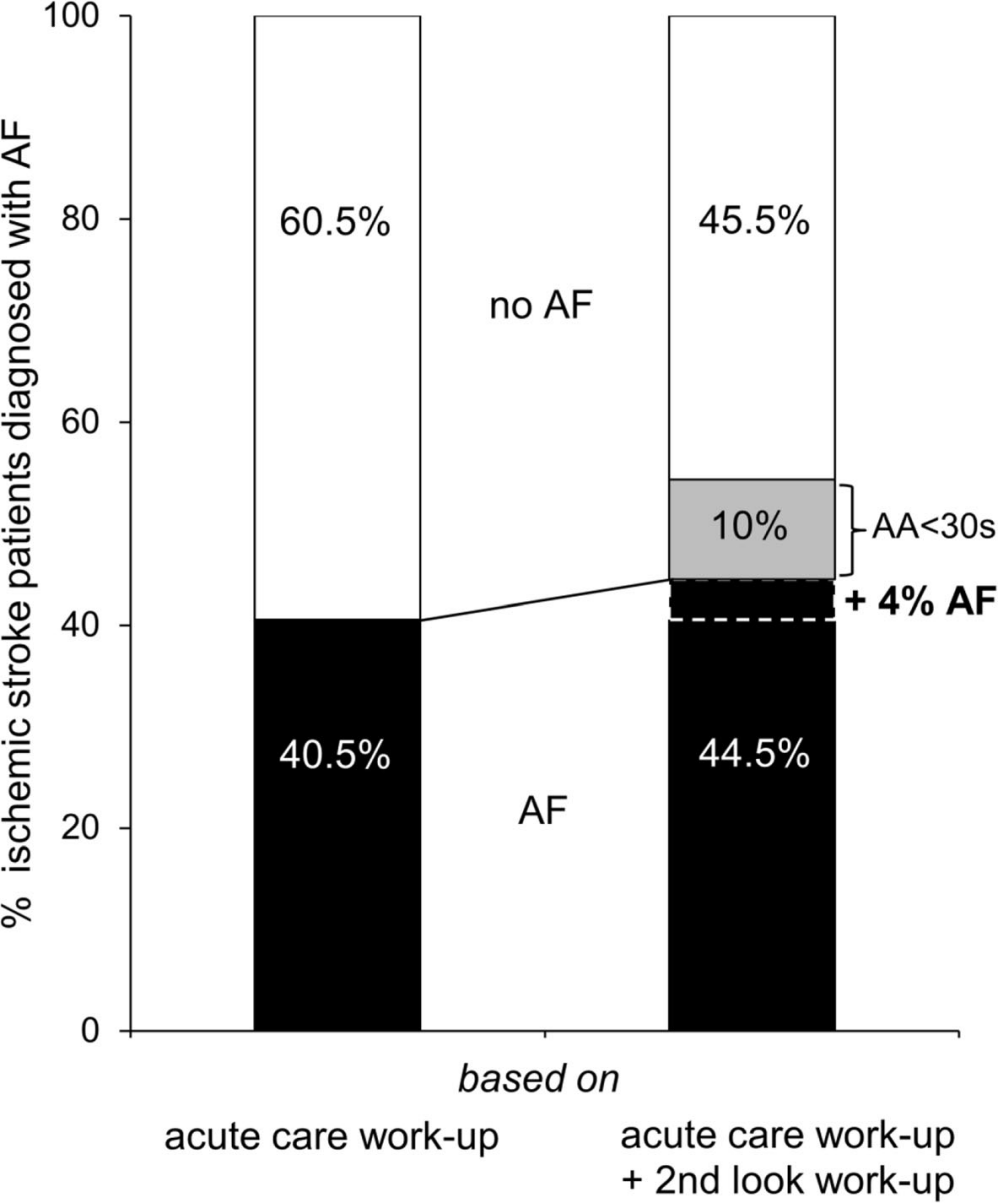
Proportion of patients with ischemic stroke (total *n* = 508) diagnosed with AF based on acute care work-up alone (left) and based on both acute care work-up and post-acute second-look ECG performed during inpatient neurorehabilitation

For 206 of the 508 patients with ischemic stroke admitted to our neurorehabilitation center, AF was already confirmed during the acute-care work-up. Details of the diagnostic work-up during acute care in referring hospital are provided in Table 2. Together with the 20 newly detected cases, the *total* rate of AF in ischemic stroke patients requiring inpatient neurorehabilitation was therefore determined as 226 out of 528 patients or 44.5% of the cohort (see Fig. 2).

**Table 2:** ECG during acute care in referring hospital

Group	Ischemic stroke admitted without diagnosis of AF		Ischemic stroke admitted with diagnosis of AF	Hemorrhagic stroke
	no AF diagnosis during re-habilitation	newly diagnosed with AF during re-habilitation		
Number of patients	282	20	206	100
Stroke unit acute care	83%	85%	85%	46%
Non-stroke unit acute care	17%	15%	15%	54%
Diagnostic work-up during acute care, as % of sample				
Standard ECG / of which resulted in AF diagnosis	71% / 0%	75% / 0%	65% / 68%	44% / 0%
Holter ECG / of which resulted in AF diagnosis	51% / 0%	50% / 0%	63% / 89%	9% / 0%
Bedside monitor ECG / of which resulted in AF diagnosis	14% / 0%	5% / 0%	11% / 90%	5% / 0%
Information missing	16%	15%	15%	50%
Etiology according to acute care diagnosis				
cardiogenic	6%	5%	90%	
arteriosclerotic	63%	65%	5%	
micro-angiopathy	9%	5%	1%	
not specified	16%	25%	4%

**Fig. 3:**
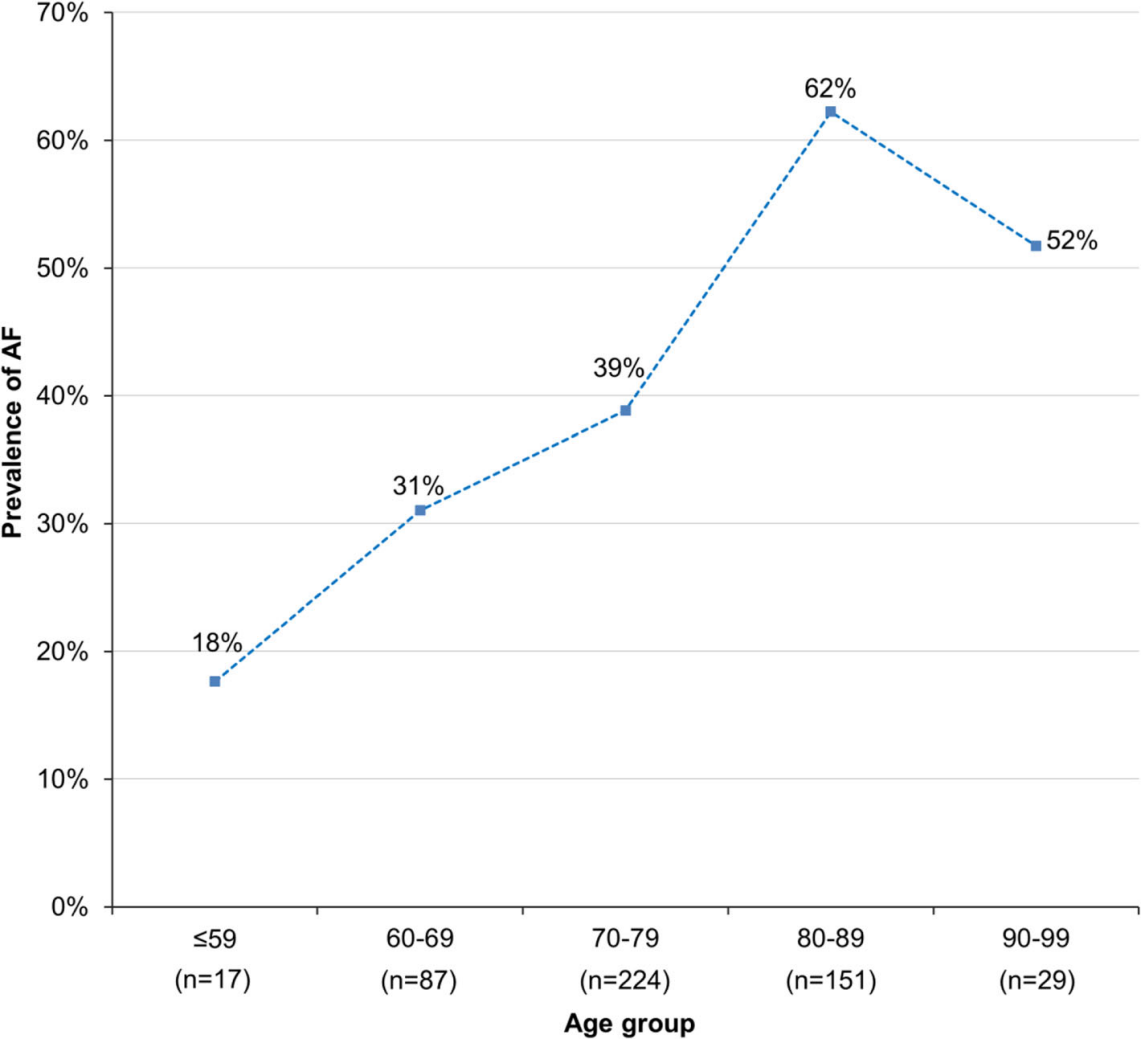
Prevalence of AF (based on both acute care work-up and post-acute second-look 24 h ECG) relative to age (x-axis) in patients with ischemic stroke requiring inpatient neurorehabilitation (number of patients per age group in brackets)

Across all ischemic stroke patients, AF (diagnosed during acute care and/or neurorehabilitation) showed a significant association with age (logistic regression with age as predictor and AF (yes/no) as independent variable; β = .05, *p* < .0001). The highest prevalence of AF was observed for patients aged 80 to 89 years and was 62% (see Fig. 3).

### Sensitivity: results of post-acute work-up in patients admitted with AF diagnosis

In the 206 patients with ischemic stroke in whom AF had been detected during acute care, second-look baseline ECG revealed AF in 138 cases (i.e. 67% of the sample). A subsequent 24-h Holter ECG confirmed AF in a further 34 patients (i.e. 16.5% of the sample). In the remaining 34 individuals (i.e. 16.5% of the sample), second-look 24-h ECG did not show AF. These results again demonstrate that (paroxysmal) AF is sometimes missed in a 24-h ECG.

### Results of post-acute 24-h ECG in the comparison group with hemorrhagic group

In patients with hemorrhagic stroke, 24-h ECG performed during post-acute neurorehabilitation revealed AF in 2 patients (i.e. 2% of cohort) and absolute arrhythmia of < 10 s in 6 patients (6%). Absolute arrhythmia of 10–30 s did not occur in this cohort.

## Discussion

### Main findings

This prospective evaluation investigated the diagnostic yield of second-look 24-h ECG in 508 consecutive patients with ischemic stroke selected on the basis of requiring inpatient neurorehabilitation. Stroke patients requiring inpatient neurorehabilitation are older and more severely affected than unselected stroke survivors. Given that both of these factors are associated with increased risk for AF [12], we postulated that stroke patients requiring inpatient neurorehabilitation constitute a high-risk cohort. Our data confirm this hypothesis. The total (combined) prevalence of AF in our neurorehabilitation cohort of ischemic stroke patients was 44.5% (226 out of 508 patients). In comparison, previously reported prevalence of AF based on 24–74 h of ECG monitoring in unselected samples of ischemic stroke or ischemic transient attack survivors typically ranged between 5 and 10% [9, 17–21].

The *added* diagnostic yield of second-look ECG work-up during post-acute care, consisting of a baseline ECG and subsequent 24-h Holter ECG, in this high-risk cohort was also impressive: AF was detected in 6.6% of ischemic stroke patients for whom acute-phase work-up found no AF. In comparison, in a cohort of unselected stroke patients who were re-assessed by second-look 24-h ECG within 6 month of their stroke in the EMBRACE control arm, the rate of newly detected AF was 3.2% [8], approximately half of that found here. Similarly, a recent prospective multicenter trial showed that prolonging acute-phase Holter ECG monitoring from 24 h to 72 h resulted in an *added* detection of 1.7% cases [9], 4–5 times less than that found in the current study. Moreover, our detection rate is five times higher than that found in unselected stroke patients re-assessed at scheduled and unscheduled visits with ECG monitoring performed at the discretion of stroke specialists in the CRYSTAL-AF control cohort [7].

### Length of arrhythmias

The diagnosis of paroxysmal AF requires the detection of an arrhythmia of 30 s or longer [22]. This definition is based on convention, but has been widely accepted. Nonetheless, observational data suggest that shorter atrial arrhythmias identify patients with AF, and that patients with such arrhythmias have an increased risk of stroke [23–25]. Therefore, in clinical practice, many patients with briefer AF episodes after cryptogenic stroke are prescribed anticoagulant therapy - particularly by neurologists [16]. For instance, the STROKESTOP mass screening for AF study in Sweden used two episodes of irregular rhythm without p-waves of 10–29 s as a criterion for anticoagulation prescription [26]. Further, other studies evaluated the usefulness of oral anticoagulation in all patients with embolic stroke of unknown origin and no positive documentation of AF in 24-h ECG where many eligible patients will have undetected atrial arrhythmias or even undetected atrial fibrillation [2, 3]. Here, we thus also reported on patients with short atrial arrhythmias. We found that an additional 3.6% of ischemic stroke patients for whom acute-care work-up showed no AF had atrial arrhythmias of 10–30 s duration and an additional 12.9% had arrhythmias of less than 10 s. All durations taken together, arrhythmias were thus found in 23%, or one in four patients, of the subsample, which corresponds well with the results of a recent meta-analysis by Sposato and colleagues on prevalence of atrial arrhythmias of any length [27]. However, their meta-analysis reviewed findings from a four-stage sequential screening including inpatient serial monitoring, ambulatory Holter ECG, as well as outpatient external and implantable loop recordings. In other words, in our high risk group, 24 h-screening provided equally high detection rates of atrial arrhythmias as extensive and invasive sequential screenings in unselected cohorts.

### Causality

Differentiation between patients with AF antecedent to stroke and those with new onset AF is not feasible. Brain lesions may trigger AF [28, 29]. Could AF in this cohort reflect an effect of brain lesion rather than of stroke cause, particularly since our patients presumably had larger lesions than unselected stroke patients? To answer this question we also assessed AF in patients with brain hemorrhages without a history of AF, as an internal control for the effect of brain lesion unrelated to cardiogenic embolism. For this group, detection rate of AF in 24-h ECG during neurorehabilitation was only 2%, i.e. three times less often than in patients with ischemic stroke. These differences did not, however, reach significance on chi-square testing for independence (*p* = 0.078). Short arrhythmia were found in 6% of hemorrhagic stroke patients and were thus also significantly rarer in hemorrhagic compared to ischemic stroke patients. These findings suggest that AF in our cohort was much more likely to be the cause for rather than a sequel of ischemic brain lesion.

### Limitations

Some of our clinical data were obtained from outside acute care institutions with variability in type and documentation of the primary diagnostic work-up. In some cases, mode of ECG assessment was not reported (see Table 2). Similarly, documentation of etiological classification of strokes was heterogeneous.

### Cost-effectiveness

Outpatient cardiac monitoring for detection of previously missed AF in a hypothetical cohort of 70-year-old stroke survivors has been found cost-effective over a range of models based on an added diagnostic yield of 4.4% [30]. Comparable results have been obtained from the Swedish system based on use of health care resources, savings of costs and lives, and improvement of quality of life [31].

Our data did not allow for a full cost-effectiveness model. However, since inpatient second-look ECG monitoring requires less incremental logistics, allows for more timely diagnosis and treatment and provides higher detection rates, we assume that second-look 24-h ECG in inpatient neurorehabilitation is even more cost-effective than outpatient monitoring.

Annual recurrence rate of up to 19% have been reported in stroke patients with AF detected after discharge [32]. Conversely, in the age group older than 75 years, anticoagulation initiated after detection of AF provides almost a 60% risk reduction of stroke relative to antiplatelet therapy [33, 34]. Given the 6% detection rate of previously missed AF in the present study, a second-look 24-h ECG during inpatient neurorehabilitation with appropriate subsequent treatment could therefore, over the course of five years, prevent one recurrent stroke for every 25 patients evaluated. The number needed to screen would be even lower if shorter duration atrial arrhythmias were included. Given that around 40% of ischemic stroke patients are referred to inpatient post-acute rehabilitation [35], the overall impact of a second-look ECG for AF would be substantial.

Inpatient neurorehabilitation allows for second-look etiological evaluation, management of medical complication, training of impaired functions and initiation of future care. However, as yet, diagnostic measures have neither been incorporated into guidelines for post-stroke neurorehabilitation, nor are they reimbursed. The present study demonstrates that secondary prevention of stroke could gain substantially by better use of the full chain of care.

## Conclusions

A simple second-look 24-h ECG in stroke survivors during inpatient neurorehabilitation identifies more patients with previously missed AF than in most other settings and should be made a standard of care.

## Data Availability

Please contact corresponding author for data requests
